# Supplementary material for: A Feasibility Study to Increase Chronic Hepatitis C Virus RNA Testing and Linkage to Care among Clients Attending Homeless Services in Amsterdam, The Netherlands
Source: Diagnostics (Basel). 2021 Jun 30;11(7):1197. doi: 10.3390/diagnostics11071197 (PMC8306529; doi:10.3390/diagnostics11071197)
Supplement: Supplementary file 1 [file diagnostics-11-01197-s001.zip › diagnostics-1221417-supplementary.pdf]

Supplementary Table S1. Prior distributions used in analysis

Assumed direction of association	Dichotomous variable OR (95% CrI)	Continuous variable OR (95% CrI)
Possibly protective	NA	0.90 (0.23-3.60)
Unknown direction	1.00 (0.25-4.00)	NA
Possibly harmful	1.50 (0.38-6.00)	NA
Probably harmful	2.00 (0.50-8.00)	NA

Abbreviations: CrI, credible interval; OR, odds ratio; NA, not applicable (i.e. not applied in analysis)
All priors based on log-normal distribution with mean ln(HR) and variance 1/2 [1].

Reference:

1. Greenland S. Prior data for non-normal priors. Stat Med. 2007 Aug 30;26(19):3578-

Supplementary Table S2. Posterior estimates of risk factors for HCV-RNA positive status (using non-informative priors*)

	**Posterior OR (95% CrI)**
Sociodemographic characteristics	
Age (per 10 year increase)[a]	0.63 (0.37-1.12)
Females (versus males)	0.96 (0.03-5.75)
Eastern European origin	10.54 (2.24-82.36)
HCV-related risk factors	
Injecting drug use (ever)	10.49 (1.91-77.08)
Injecting drug use (last 30 days)	11.53 (1.91-66.84)
Ever sharing needles or syringes	6.80 (0.81-36.95)
Ever sharing straws/crack pipe	2.06 (0.38-16.58)
Ever sharing razors/toothbrush	0.75 (0.02-5.67)
Procedures[b] in HCV endemic country	0.33 (0.01-2.54)
Tattoo/piercing in nonregistered location[c]	0.46 (0.02-3.40)
Sexual behavior	
Condomless anal sex	<0.01 (<0.01-1.52)
Sharing of sex toys	<0.01 (<0.01-2.36)

Abbreviations: OR, odds ratio; CrI, credible interval.

*Non-informative priors (i.e. those that bear minimal influence when estimating the posterior distribution) were log-normal distributions with mean ln(1) and variance 100.

Supplementary Table S3. Prior and posterior estimates of risk factors for HCV-RNA positive status (using priors with weaker effects)

	**Posterior OR (95% CrI)**	**Posterior OR (95% CrI)**
Eastern European origin	1.50 (0.38-6.00)	2.79 (1.03-7.75)
Injecting drug use (ever)	1.50 (0.38-6.00)	3.21 (1.14-9.41)
Injecting drug use (last 30 days)	1.50 (0.38-6.00)	3.42 (1.09-10.37)

Abbreviations: CrI, credible interval; OR, odds ratio.

ORs and 95% CrI were obtained from univariable Bayesian exponential survival models.

Supplementary Table S4. Risk factors for HCV-RNA positive status, without using a Bayesian approach
(to illustrate that using ordinary regression is not appropriate due to sparse data issues)

	OR (95% CI)
Sociodemographic characteristics	
Age (per 10 year increase)[a]	0.61 (0.36-1.10)
Females (versus females)	1.20 (0.14-9.95)
Eastern Europe origin	15.38 (2.79-84.72)
HCV-related risk factors	
Injecting drug use (ever)	13.33 (2.28-78.14)
Injecting drug use (last 30 days)	19.14 (3.26-112.56)
Ever sharing needles/syringes	13.30 (1.96-90.53)
Ever sharing straws/crack pipe	2.25 (0.40-12.67)
Ever sharing razors/toothbrush	0.71 (0.08-6.54)
Procedures[b] in HCV endemic country	0.40 (0.05-3.52)
Tattoo/piercing in nonregistered location[c]	0.73 (0.08-6.31)
Sexual behavior	
Condomless anal sex	--
Sharing of sex toys	--

[a]Age was centered at 18 years.
[b]Includes any medical procedure, blood transfusion, tattooing, piercing, and/or dental procedure.
[c]Taking place in the Netherlands.
-- OR could not be calculated because the given factor was not found in one of the HCV-RNA groups.
CrI, credible interval; HCV, hepatitis C virus; OR, odds ratio; IQR, interquartile range.

Supplementary Table S5. The presence of HCV related risk factors and sexual behavioral factors for each HCV RNA positive individual

	**HCV RNA positive individuals (N=7)**							**Total N (presence = yes), %**
HCV related risk factor	#1	#2	#3	#4	#5	#6	#7	
Injecting drug use (ever)	1	M	1	1	0	0	1	4, 67%
Injecting drug use (last 30 days)	1	M	1	1	0	0	0	3, 50%
Ever sharing needles/syringes	0	M	1	0	0	0	1	2, 33%
Ever sharing straws/crack pipe	1	M	0	1	1	0	1	4, 67%
Ever sharing razors/toothbrush	0	M	0	0	0	0	1	1, 17%
Procedures[b] in HCV endemic country	1	M	0	0	0	0	0	1, 17%
Tattoo/piercing in nonregistered location	0	0	0	0	0	0	1	1, 14%
Sexual behavior								
Condomless anal sex	0	M	0	0	0	0	0	0, 0%
Sharing of sex toys	M	M	0	0	M	0	0	0, 0%

1: variable is present, 0: variable is not present, M: variable is missing in this individual.
